# Human Behavior Recognition via Hierarchical Patches Descriptor and Approximate Locality-Constrained Linear Coding

**DOI:** 10.3390/s23115179

**Published:** 2023-05-29

**Authors:** Lina Liu, Kevin I-Kai Wang, Biao Tian, Waleed H. Abdulla, Mingliang Gao, Gwanggil Jeon

**Affiliations:** 1 College of Electrical and Electronic Engineering, Shandong University of Technology, Zibo 255000, China; 2 Department of Electrical, Computer, and Software Engineering, Faculty of Engineering, The University of Auckland, 20 Symonds St, Auckland 1010, New Zealand; 3 Science and Technology Cooperation and Exchange Center of Zouping, Zouping 256200, China; 4 Department of Embedded Systems Engineering, Incheon National University, Incheon 22012, Republic of Korea

**Keywords:** human behavior recognition, energy image species, hierarchical patches descriptor, approximate locality-constrained linear coding algorithm

## Abstract

Human behavior recognition technology is widely adopted in intelligent surveillance, human–machine interaction, video retrieval, and ambient intelligence applications. To achieve efficient and accurate human behavior recognition, a unique approach based on the hierarchical patches descriptor (HPD) and approximate locality-constrained linear coding (ALLC) algorithm is proposed. The HPD is a detailed local feature description, and ALLC is a fast coding method, which makes it more computationally efficient than some competitive feature-coding methods. Firstly, energy image species were calculated to describe human behavior in a global manner. Secondly, an HPD was constructed to describe human behaviors in detail through the spatial pyramid matching method. Finally, ALLC was employed to encode the patches of each level, and a feature coding with good structural characteristics and local sparsity smoothness was obtained for recognition. The recognition experimental results on both Weizmann and DHA datasets demonstrated that the accuracy of five energy image species combined with HPD and ALLC was relatively high, scoring 100% in motion history image (MHI), 98.77% in motion energy image (MEI), 93.28% in average motion energy image (AMEI), 94.68% in enhanced motion energy image (EMEI), and 95.62% in motion entropy image (MEnI).

## 1. Introduction

The proliferation of interconnected devices has led to the scenario of the Internet of Everything (IoE), which enables many intelligent and context-aware applications. Human behavior recognition, which is broadly applied to intelligent surveillance, human–machine interaction, video retrieval, etc. [1,2,3], has recently attracted more attention in computer vision. At present, most of the research on behavior recognition is based on video sequence analysis. Despite the significant progress made in this area, this remains a complex and challenging task. There are significant variations caused by subject behavior, viewpoint variations, occlusions, camera motion, cluttered background, the similarity between different behaviors, and even movement variability of the same behavior. Due to the aforementioned factors, researchers have put forward many different countermeasures.

Human behavior recognition contains two main tasks, namely behavior feature extraction and behavior pattern recognition. Feature extraction is the dominant step. With a given human behavior recognition framework, the performance of human behavior recognition depends on the quality of the feature extraction [4,5]. Human behavior recognition approaches based on vision can be separated into two categories: the traditional artificial-feature-based approach and the learning-feature-based approach [4,6]. The artificial features are dependent on the predesigned feature detectors and descriptors, which are relatively simple and easy to implement. However, they are difficult to interpret intuitively and have the problem of low recognition accuracy. The learning-feature-based approach is divided into two major categories, some of these approaches being sparse-representation and dictionary-learning-based methods, and others being deep-learning-based models. Dictionary learning employs the sparse representation of the input data, which is applicable to image or video-based classification tasks. Although the sparse-representation and dictionary-learning-based approaches have obtained good performance on several public datasets, rapidly constructing an effective dictionary-learning model for behavior recognition remains challenging. As it needs to solve norm optimization problems repeatedly during the model optimization process, this process has high computation cost and execution time.

Meanwhile, with the boom in artificial intelligence, deep learning has made remarkable achievements in the computer vision area. In many real-world applications, there may not exist enough large-scale datasets for training a deep-learning model. Therefore, especially for small-scale datasets, it is still a challenge to improve the recognition accuracy and robustness. Some of the problems include:

(1) Traditional hand-crafted representation-based features are difficult to interpret intuitively and have the problem of low recognition accuracy;
(2) Learning an effective dictionary-learning model is computationally expensive and time-consuming;
(3) For small-scale datasets, it is still a challenge to improve the recognition accuracy and robustness.

To address the aforementioned constraints, in this paper, a unique human behavior recognition approach is proposed based on a hierarchical patches descriptor (HPD) and ALLC algorithm. The main contributions of this article are as follows:

(1) Five energy image species are utilized to describe human behavior in a global manner. These are statistical features based on motion information. Moreover, an HPD is constructed to obtain detailed local feature descriptions for recognition. Combining local features with global features can better describe behavioral features, which can improve recognition accuracy.
(2) The proposed method is based on the ALLC algorithm for fast coding, which is computationally efficient because it has a closed-form analytical solution and it does not need to solve the norm optimization repeatedly.
(3) We demonstrate the superior performance of the proposed method in comparison with state-of-the-art alternatives by conducting experiments on both Weizmann and DHA datasets.

The remainder of this paper is organized as follows: Related work is presented in Section 2. The framework of the proposed approach, human behavior feature extraction, and a human behavior recognition scheme is presented in Section 3. Section 4 analyses experimental results and Section 5 presents the discussion. The paper is concluded in Section 6.

## 2. Related work

### 2.1. Traditional Artificial-Feature-Based Approach

The traditional artificial-feature-based approach is dependent on the predesigned feature detectors and descriptors, such as the bag-of-words (BoW) model [7], scale-invariant feature transform (SIFT) [8,9] and weighted hierarchical features [10], histogram of oriented gradients (HoG) [11,12] and pyramid histogram of oriented gradients (PHOG) [13], and local binary pattern (LBP) [14]. These features are relatively simple and easy to implement, but are difficult to interpret intuitively and have the problem of low recognition accuracy.

### 2.2. Learning-Feature-Based Learning Approach

Unlike the handcrafted-feature-based approaches, with the help of the concepts of a trainable feature extractor and classifier, feature-learning-based approaches can automatically learn features from the input data. Some of these approaches are based on sparse-representation and dictionary learning, and others are based on deep-learning models. Dictionary learning employs the sparse representation of the input data, which is applicable to image- or video-based classification tasks. Dictionary learning has been widely employed in computer vision areas, such as image classification [15,16,17,18,19] and action recognition [13,20,21,22]. Wright et al. [15] were one of the pioneers that used sparse representation for face recognition and achieved good results. The sparse coding [16] and locality-constrained linear coding (LLC) algorithms were widely used to deal with image classification [18], multiview facial expression recognition [19], and view-invariant action recognition [23]. Wang et al. [20] proposed to divide the 3D skeleton sequence into multiple non-interrelated sub-sequences, and used the coordinated representation of the motion density trajectories of the sub-sequences for behavior recognition.

Aiming to deepen the image sequence, Gao et al. [22] proposed a multi-feature mapping and dictionary-learning model (MMDLM) to obtain the correlation of different features, where MMDLM is a typical multi-modality dictionary-learning algorithm for feature fusion. The multi-modality joint representation and recognition (MMJRR) [12] is also a typical multi-modality algorithm for action recognition. Moreover, an RGBD action recognition approach based on a collaborative sparse representation (CSR) learning model was proposed in [22], where BoW features were extracted for RGB and depth modality, respectively. Then, they were weighted together by the CSR learning algorithm, and the collaborative reconstruction error was applied for classification.

Meanwhile, with the boom in artificial intelligence, deep learning has made remarkable achievements in the computer vision area. In particular, convolutional neural network (CNN)- and recurrent neural network (RNN)-based approaches have been widely used in human behavior feature extraction [24,25,26,27]. Wang et al. [24] proposed a three-stream CNN to learn behavior descriptors by feeding weighted layer depth motion maps to the network. Sharif et al. [25] proposed a hand-crafted and deep CNN feature fusion and selection strategy, and HOG features as the input of the CNN model for recognition. Bhatt et al. [26] summarized CNN variants for computer vision from five aspects: history, architecture, application, challenges, and future scope. Patel et al. [27] proposed a dimension-based generic convolution block for object recognition. Due to overfitting caused by the lack of training data, learning an effective deep neural network for action recognition remains a challenge. Therefore, data augmentation [24] and synthetic depth images [25] were used to reduce the possibility of overfitting. The introduction of some large-scale RGBD-based datasets [28,29,30] made it possible to develop more effective action recognition approaches based on deep learning.

Inspired by the above research, this work combines an artificial-feature-based approach and a feature-learning-based approach to describe the human behavior feature in a more detailed manner. Furthermore, a fast-coding method is utilized to improve the efficiency of recognition.

## 3. The Proposed Methods

### 3.1. Framework of the Proposed Human Behavior Recognition Approach

Aimed at improving the accuracy and robustness of human behavior recognition, a unique human behavior recognition approach based on HPD and ALLC is proposed. In the proposed technique, five energy image species for each human behavior video sequence are first calculated to describe human behavior in a global manner. The energy image species include motion energy image (MEI) and motion history image (MHI) [31], average motion energy image (AMEI), enhanced motion energy image (EMEI), and motion entropy image (MEnI) [32]. However, these energy image species cannot describe the local human behavior in detail, and HPD is proposed to analyze the energy image species at different scales for describing the local details of human behavior. Thus, we encode the HPD by using an ALLC algorithm for fast coding to acquire effective coding for human behavior recognition.

**Figure 1:**
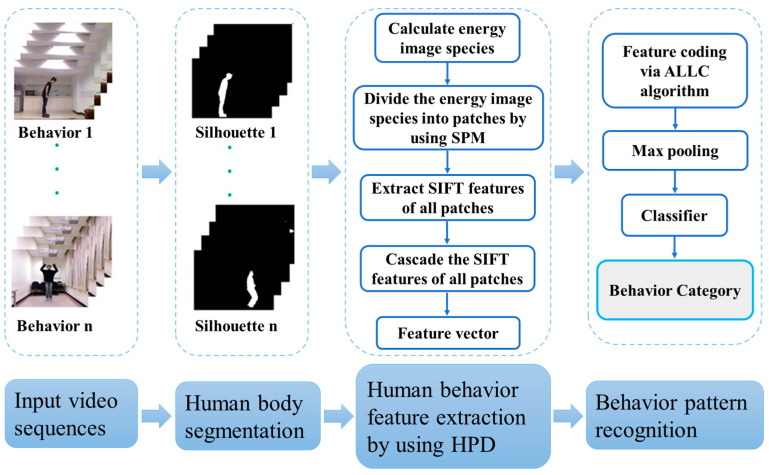
Framework of the proposed human behavior recognition approach.

The framework of the proposed human behavior recognition approach is illustrated in Figure 1. The overall process consists of three major steps: human body segmentation, human behavior feature extraction, and behavior pattern recognition.

The details of each individual step are as follows.

(1) Human body segmentation. In the input video sequences, there often exists a large amount of background information, which significantly reduces the computation efficiency and affects the human motion feature extraction. Thus, segmentation is an essential step to ensure that critical behavior information can be retained while unnecessary background information can be removed. In this paper, human behavior recognition is targeted at the whole body behavior, instead of the actions of specific human body parts. Therefore, the human body silhouette is segmented from the background as the input data for the feature extraction step.
(2) Human behavior feature extraction. To describe the human behavior information in detail, a combined strategy of global and local feature extraction is utilized in the paper. For each video sequence, several energy image species of the human body silhouette images are calculated as global feature descriptors of the human behavior. The advantage of this method is that it can describe the global human behavior information well in a statistical manner by using one image per video, which can greatly reduce the computational load of local feature extraction in the following processes. However, it cannot express the local human behavior information well. Therefore, after calculating each energy image species, an HPD is constructed to describe the local feature information of the targeted human behavior, which contains three steps.

Firstly, the energy image species is divided into patches on different resolutions by adopting the spatial pyramid matching (SPM) algorithm. Secondly, the BoW model with spatial–temporal features is employed to analyze the energy image species at different scales for local descriptions of human behavior. In view of obtaining local features that are scale-invariant, the SIFT features of all patches are extracted, which will generate numerous features that can densely cover the image in the whole scale and location range, which is beneficial to describe the local human behavior information. Finally, the SIFT features of all patches are cascaded together to form a vector for recognition.

(3) Behavior pattern recognition. After extraction of human behavior features from the video sequences, different human behaviors are learned individually from the training video sequences of each class by using the ALLC algorithm and max-pooling. Each testing video sequence is then attributed to a predefined class according to its corresponding feature. At this stage, the HPD feature vectors are encoded together by the ALLC algorithm, which is a simple, yet effective, fast coding algorithm.

Since the ALLC algorithm has better constructability and local smooth sparsity, the correlations between similar descriptors can be obtained easily by ensuring similar patches have similar codes, which is beneficial for human behavior recognition. In addition, it has an analytical solution and does not need to solve the norm optimization repeatedly, as in a sparse coding algorithm. Therefore, it has higher computational efficiency and needs less storage space in the process of objective function optimization, making it an effective and simple fast coding algorithm.

The coding results of all HPD feature vectors are in matrix form, which makes it difficult to construct eigenvectors for recognition. Therefore, it is necessary to pool all codes together and cascade them together to form a final feature vector for recognition. Considering that max-pooling almost always performs better than average pooling, especially with a linear SVM [33,34], max-pooling is used in the proposed approach.

### 3.2. Human Behavior Feature Extraction

#### 3.2.1. Environmental Modelling and Human Body Segmentation

**Figure 2:**
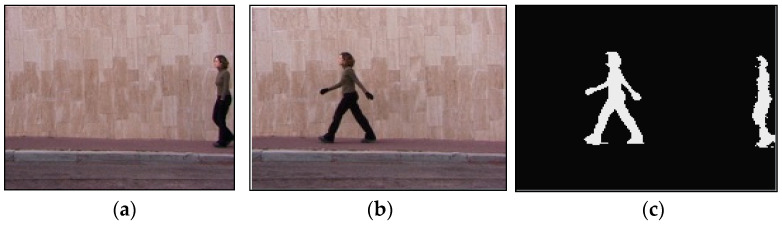
Pedestrian detection results in a single-frame image. (**a**) The initial frame, (**b**) the current frame, and (**c**) the detection result of ghost area.

Human body segmentation is the basis of behavior recognition, and it aims to extract the body silhouette from an image sequence. In this paper, the background difference method [35] is employed to extract the human body silhouette. This method assumes that the background changes slowly or tends to be stationary, but in reality, there often exist factors such as light changes, background disturbances, and camera jitter. Therefore, it is necessary to model the background. However, if the initial frame used for modeling contains a moving target, the previous foreground target will be taken as background in the foreground determination step, which will lead to the so-called ghost area appearing in the pedestrian detection results of the current frame, as shown in Figure 2.

To remove the ghost area, the VIBE background modelling method is adopted to extract the moving human target contour [36]. VIBE has the characteristics of less computational cost, fast speed, and less memory. By randomly selecting images, the temporal correlation can be improved, and the actual scene can be better coped with. By randomly selecting neighborhood locations, the spatial correlation can be improved, and the camera jitter can be dealt with, thus, the ghost area can be eliminated as soon as possible.

**Figure 3:**
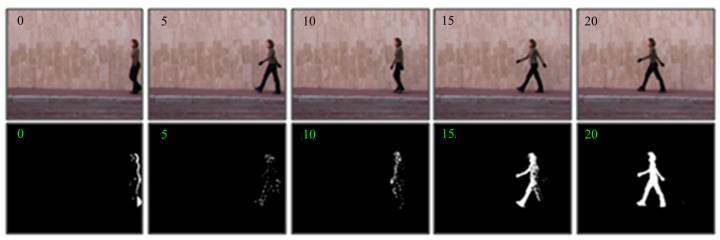
Pedestrian detection results of ghost area elimination by background updating strategy, where the numbers represent the number of frames.

Figure 3 shows the pedestrian detection results in the 0–20th frame (every 5 frames) of a walking video by background-updating strategy. In Figure 3, we can observe that those ghost areas remain in the contours of target detection in subsequent frames since the initial frame contains a moving target. However, the ghost area residues gradually disappear with the updating strategy. By the 20th frame, the outline of the human body has become very clear. Therefore, VIBE can utilize the spatial propagation advantages of the pixels to gradually diffuse the background model outward and quickly eliminate the ghost areas.

#### 3.2.2. Calculation of the Energy Image Species

Energy image species is one type of global feature, which is commonly used to statistically represent the spatial–temporal information of behavior. It mainly targets object contour images and has the advantages of simple calculation and not being sensitive to the background and movement time [31]. In this paper, five energy image species are utilized to represent human behavior, namely, MEI, MHI [31], AMEI, EMEI, and MEnI [32]. Although the five energy image species are all global descriptions, they are still slight differences because they are focused on different contents. The MEI and MHI focus on the change of human motion with time and the motion that happened at an earlier time, respectively. AMEI focuses on the overall movement by using binary contours, while EMEI is extracted to highlight the dynamic parts, and MEnI is defined by computing the Shannon entropy of the average motion energy image, trying to reflect the dynamic process from a microscopic perspective.

Let $I_{seq}(x,y,t)$ denote an image sequence and $D_{dif}(x,y,t)$ represent a binary image sequence, which indicates the motion regions of $I_{seq}(x,y,t)$, and can be calculated by image differentiating, i.e., $D_{dif}(x,y,t)=\;$$I_{seq}(x,y,t+1)$$-I_{seq}(x,y,t)$, where t, 1≤t≤N represents the *t*-th frame, and N is the duration of the considered image sequence. Specific calculations of the five energy image species are as follows:

(1) MEI and MHI: The binary MEI $E_{MEI}(x,y,t)$ and MHI $E_{MHI}(x,y,t)$ can be calculated by Equations (1) and (2), respectively.

(1) $$E_{MEI}(x,y,t)=\overset{\tau -1}{\underset{i=0}{\cup }}D_{dif}(x,y,t-i).$$

(2) $$E_{MHI}(x,y,t)=\left\{\begin{array}{cc} \tau , & \mathrm{if}\;D_{dif}(x,y,t)=1\\ \max(0,E_{MHI}(x,y,t-1)-1),\; & otherwise \end{array}\right..$$

where τ is the motion duration, which is crucial in defining the temporal range of behavior.

(2) AMEI, EMEI, and MEnI: For the whole motion sequence of *N* frames, the average value of the binary contour is calculated as AMEI, which is shown in Equation (3).

(3) $$E_{AMEI}(x,y)=\frac{1}{N}\sum_{t=1}^{N}I_{seq}(x,y,t)$$

EMEI is calculated by:

(4) $$E_{EMEI}(x,y)=\frac{1}{N}\sum_{t=1}^{N}\left\|I_{seq}(x,y,t)-E_{AMEI}(x,y)\right\|.$$

MEnI can be computed by:

(5) $$\begin{array}{cc} E_{MEnI}(x,y)= & -\frac{1}{N}\sum_{t=1}^{N}I_{seq}(x,y,t)\times \log_{2}(\frac{1}{N}\sum_{t=1}^{N}I_{seq}(x,y,t)+\lambda )\\ & -(1-\frac{1}{N}\sum_{t=1}^{N}I_{seq}(x,y,t))\times \log_{2}(1-\frac{1}{N}\sum_{t=1}^{N}I_{seq}(x,y,t)+\lambda ) \end{array}$$

where λ is a small positive parameter, which is introduced to avoid the zero value for a logarithmic function.

**Figure 4:**
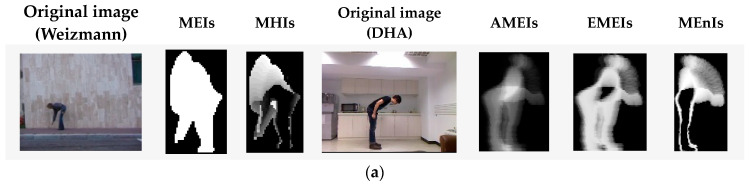

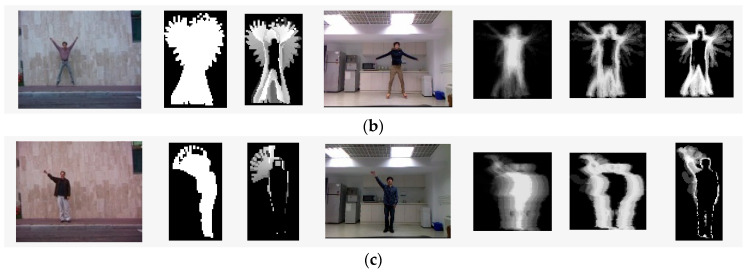
Some samples of the energy image species on the Weizmann and DHA datasets. (**a**) Bend behavior, (**b**) jack behavior, and (**c**) one-hand wave (wave1) behavior.

As can be seen from Figure 3, the object contour images have an obvious black background. Therefore, the energy species of such an image will also have a black background, which does not express any behavior information and varies in size depending on the silhouettes of different performers. When we extract features from such energy species, it will not only increase the computation load but also affect the recognition results. Therefore, to remove the black background area, we extract the minimum bounding rectangle of the target contour region, i.e., the region of interest (ROI). Several samples of the energy image species on the Weizmann and DHA datasets are shown in Figure 4.

#### 3.2.3. Construction of the Hierarchical Patches Descriptor (HPD)

By comparing with the original motion images in the leftmost column of Figure 4, we can see that the energy image species can represent the motion information in a global manner for most behaviors, such as the motion of body parts, the action area of the trunk, and the motion range of limbs. However, it cannot describe the details of local motion information very well. Taking one-hand wave behavior as an example, we find that the static trunk is clearly presented using AMEI and EMEI. In contrast, the waving hand and arm parts are shown as a vague shape area, which is very likely to lead to confusion with other similar behavior, such as a typical motion in tai chi. Therefore, it is necessary to extract local detailed features for more accurate recognition.

Recently, BoW has been one of the most successful methods used to describe the detailed features of images. The investigation of many extension methods of BoW shows that SPM [37] reports the most successful results. Therefore, in this paper, an HPD is constructed by using the SPM-based BoW model, and the algorithm flow is shown in Algorithm 1.
**Algorithm 1 Construction Process of HPD****Input** Energy image species $E_{MEI}(x,y,t)$, $E_{MHI}(x,y,t)$, $E_{AMEI}(x,y)$, $E_{EMEI}(x,y)$,
**and**
$E_{MEnI}(x,y)$;
**Output** HPD feature vector X:
**Step 1:** Obtain SIFT descriptors. For each energy species, the SIFT descriptors of 31×31 patches calculated over a grid with a spacing of 16 pixels are extracted from each key point or patch as local features. This is realized by using a difference-of-Gaussian function: 
$D_{sift}(x,y,\sigma )=(G(x,y,k\sigma )-G(x,y,\sigma ))*E(x,y)=L(x,y,k\sigma )-L(x,y,\sigma )$. 
$\mathrm{where}\;G(x,y,\sigma )=\frac{1}{2\pi \sigma^{2}}\exp^{-\frac{\left(x^{2}+y^{2}\right)}{2\sigma^{2}}}$.
**Step 2:** Generate a codebook with *M* channels by sparse coding [8]. To improve the computational efficiency, the *K*-means clustering method can be used to compute the cluster centers.
**Step 3:** Encode the descriptors. Each SIFT descriptor is encoded into a code vector with codewords in the codebook and each descriptor is transferred to an $R^{M}$ code.
**Step 4:** Spatial feature pooling.  (a)Segment the image into finer spatial subregions by using SPM method; (b)Construct a histogram by pooling multiple codes of each subregion together after averaging and normalizing operations; (c)Cascade the histograms of all patches in different spatial pyramid segmentation levels to form the HPD feature vector X.Get the HPD feature vector.

**Figure 5:**
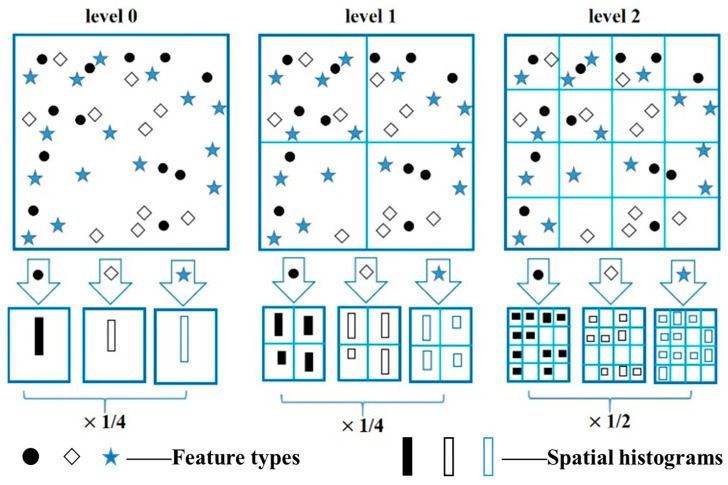
A simple schematic of structuring a three-level spatial pyramid.

Figure 5 shows a simple schematic of structuring a three-level spatial pyramid. We assume that the energy image species have three feature types, expressed in circles, rhombuses, and stars. First, the image is divided into three different levels of scale. Second, the features that fall in each spatial bin are counted for each level of the scale channel. Last, on the basis of a spatial-pyramid match kernel function, each spatial histogram is weighted together; that is

(6) $$K^{L}{\left(X,Y\right)}^{T}=\frac{1}{2^{L-l}}I^{0}+\sum_{l=0}^{L-1}\frac{1}{2^{L-l+1}}I^{l}.$$

The spatial-pyramid match kernel is a Mercer kernel, which allows processing of Gaussian variables.

From Figure 5, we can see that the image is segmented into finer spatial subregions, and then the histograms of each subregion are computed as the local features. Generally, $2^{l}\times 2^{l}$ (where l=0,1,2) sub-regions are typically used. In this case, for L segmentation levels and *M* channels, the dimensionality of the final feature vector for human behavior recognition is

(7) $$Dim_{final}=M\times \sum_{l=0}^{L}4^{L}=M\times \frac{1}{3}\times (4^{L+1}-1).$$

### 3.3. Human Behavior Recognition Scheme Based on LLC Algorithm

The SPM method utilizes the vector-quantization (VQ) coding strategy for coding, whose code has only non-zero coefficients following the non-zero constraint condition. To improve its scalability, Yang et al. [16] proposed the sparse-coding-based SPM (ScSPM) approach, where a sparse-coding algorithm was used to a encode nonlinear code. Yu et al. [38] proposed a local coordinate coding algorithm and verified that locality is more critical than sparsity under certain assumptions. Although both coding algorithms have achieved superior performance on several benchmarks, they all need to solve the $\ell_{1}$ norm optimization, which leads to a higher computational expense. Based on this knowledge, in this paper, we employ the ALLC algorithm, which has an analytical solution and its computational cost efficiency is lower than the sparse coding and local coordinate coding. In this section, the recognition scheme based on the ALLC algorithm will be introduced in detail.

#### 3.3.1. Problem Formulation

Let $X=[x_{1},x_{2},\cdots ,x_{N}]\in R^{D\times N}$ represent a set of local features with *D* dimensionality, which is extracted from energy image species; $B=[b_{1},b_{2},\cdots ,b_{M}]\in R^{D\times M}$, $b_{j}\in R^{D\times 1}$ denote a codebook with *M* codewords; and $C=[c_{1},c_{2},\cdots ,c_{N}]\in R^{M\times N}$, $c_{i}\in R^{M\times 1}$ represent the coding vector for feature X based on codebook B. The purpose of feature coding is to obtain the coding vector C by using different coding algorithms.

For most coding algorithms, only a part of codewords will be chosen for feature representation, and its coefficients are non-zero. However, most codewords are not chosen, and their corresponding coefficients are equal to zero. Therefore, the coding vector C is usually sparse.

#### 3.3.2. The LLC Algorithm

The traditional SPM algorithm uses the VQ coding method, and the coding vector C is obtained by finding the constrained least squares fitting solution. The objective function is:

(8) $$<C>=\underset{C}{\arg\min}\sum_{i=1}^{N}{\left\|x_{i}-Bc_{i}\right\|}^{2},\;s.t.{\left\|c_{i}\right\|}_{\ell_{0}}=1,{\left\|c_{i}\right\|}_{\ell_{1}}=1,\;c_{i}\geq 0,\forall i.$$

where the cardinality constrained condition ${\left\|c_{i}\right\|}_{\ell_{0}}=1$ expresses that each coding vector $c_{i}$ contains only one non-zero element, corresponding to the quantitative ID of $x_{i}$. By searching for the nearest neighbor of its neighborhood, the single non-zero element can be obtained. The non-negative constrained term $\;{\left\|c_{i}\right\|}_{\ell_{1}}=1,\;c_{i}\geq 0$ denotes that the coding weight of $x_{i}$ is 1.

To reduce the vector loss of the VQ algorithm, the cardinality constraint condition ${\left\|c_{i}\right\|}_{\ell_{0}}=1$ can be relaxed by utilizing the sparse regularization term, and its objective function is rewritten as

(9) $$<C>=\underset{C}{\arg\min}\sum_{i=1}^{N}{\left\|x_{i}-Bc_{i}\right\|}^{2}+\lambda {\left\|c_{i}\right\|}_{\ell_{1}},\;s.t.\left\|b_{m}\right\|\leq 1,\;\forall m=1,2,\cdots ,M.$$

where the sparse constrained term has three functions: (1) due to the codebook being over-complete, i.e., M>D, it is necessary to add an $\ell_{1}$ regularization term to make sure of the uniqueness of solution for the under-determined system; (2) it allows the obtained representation to acquire a salient pattern of local descriptors; and (3) compared with VQ algorithm, the quantization error is reduced.

According to the suggestion of the local coordinate coding algorithm, the locality is more significant than sparsity. Therefore, the LLC algorithm utilizes the locality-constrained term to replace the sparsity constrained term in Equation (9), and its objective function can be written as:

(10) $$<C>=\underset{C}{\arg\min}\sum_{i=1}^{N}({\left\|x_{i}-Bc_{i}\right\|}^{2}+\lambda \left\|d_{i}\right.⊙{\left.{\;c}_{i}\right\|}^{2}),\;s.t.\;1^{T}c_{i}=1,\;\forall i.$$

where $1\in R^{M\times 1}$ is a column vector with all elements as ones, ⊙ expresses an element-wise multiplication operator, and $d_{i}\in R^{M}$ denotes a locality adaptor, and is calculated by Equation (11),

(11) $$d_{i}=\exp(\frac{D_{dis}(x_{i},B)}{\sigma }).$$

where $D_{dis}(x_{i},B)={\left[D_{dis}(x_{i}-b_{1}),\cdots ,D_{dis}(x_{i}-b_{M})\right]}^{T}$ and $D_{dis}(x_{i}-b_{j})$ express the Euclidean distance between $x_{i}$ and each codeword and σ is a tune parameter to adjust the speed of weight decay. Moreover, compared with sparse coding and local coordinate coding, the constraint condition $1^{T}c_{i}=1$ of LLC is more crucial than sparsity, which follows the shift-invariant requirements.

The LLC algorithm has a closed-form analytical solution

(12) $${\tilde{c}}_{i}=(C_{i}+\lambda diag(d_{i}))\backslash 1.$$

(13) $$c_{i}=c_{i}/1^{T}c_{i}.$$

where $c_{i}=(B-1x_{i}^{T}){\left(B-1x_{i}^{T}\right)}^{T}$ is a covariance matrix.

#### 3.3.3. ALLC Algorithm for Fast Coding

In the process of solving object function (10), a local coordinate system is constructed on the local basis of each descriptor. Moreover, without solving the objective function (10) directly, the *K-*nearest neighbors (where K<D<M) of $x_{i}$ in the codebook can be simply used as the local bases $B_{i}$, then the coding vector C is computed by solving a much smaller linear system, and its objective function is

(14) $$<C>=\underset{C}{\arg\min}\sum_{i=1}^{N}{\left\|x_{i}-c_{i}{\tilde{B}}_{i}\right\|}^{2},\;s.t.\;1^{T}c_{i}=1,\;\forall i.$$

**Figure 6:**
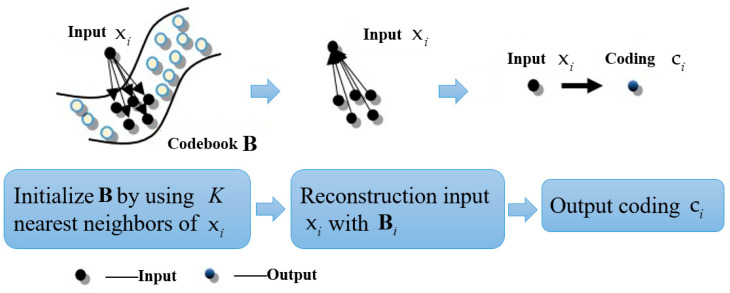
The coding process of ALLC algorithm.

Because $B_{i}$ is the K-nearest neighbor code-word for $x_{i}$, and K≪M, the approximate algorithm can reflect the locality and sparsity simultaneously. In addition, the computational complexity declined from $O(M^{2})$ to $O(M+K^{2})$, which greatly reduces the computation cost. Its coding process is illustrated in Figure 6.

#### 3.3.4. Max-Pooling

The ALLC algorithm is used to encode all patches on each level of the SPM in matrix form, which makes it difficult to construct eigenvectors. Therefore, it is necessary to pool all codes and normalize them to form a final feature vector for behavior recognition. In this paper, the max-pooling method is used, which is as follows:

(15) $$c_{out}=\max(c_{in1},\cdots ,c_{in2}).$$

where the max function is pooled in rows and the dimension of the returned vector is the same as $c_{in1}$. Moreover, the pooling feature is normalized by $\ell_{2}$ norm:

(16) $$c_{out}=c_{in}/{\left\|c_{in}\right\|}_{2}.$$

## 4. Experimental Results

### 4.1. Experimental Settings and Descriptions

The experiments reported in this section were conducted on two public human behaviour datasets, namely, the classical Weizmann dataset [39], and DHA dataset [40]. Different from the Weizmann dataset, the DHA dataset is more challenging. It contains RGB and depth data, with more variations in background, illumination fluctuations, and behavior complexity, and it is a multi-modality dataset. The details are as follows:

(1) Weizmann dataset: The Weizmann dataset consists of 10 human behavior categories, every behavior was completed by nine performers in a similar environment. Each video sequence has a different length. Following the database instructions of literature [7,41], nine behaviors were selected for MEI and MHI, which were bend, jump, jack, side, run, walk, skip, wave1 (one-hand wave), and wave2 (two-hand wave).
(2) DHA dataset: The DHA dataset contains 23 categories of human behavior (e.g., bend, jump, pitch, and arm-swing), where every behavior contains 21 performers (12 males and 9 females). The duration of the video sequences also varies. Following [10] and the database instructions, 14 behaviors were selected for MEnI, including bend, jump, jack, run, skip, walk, side, wave1, wave2, side-box, arm-swing, tai chi, and leg-kick, and 17 behaviors were selected for AMEI and EMEI, including bend, jump, jack, pjump, run, walk, skip, side, wave1, wave2, arm-swing, leg-lick, front-lap, side-box, side-box, rod-swing, and tai chi.

For convenience of comparison, the leave-one-video-out evaluation strategy was adopted to assess the approach performance. The proposed approach was compared with some existing techniques mainly on three aspects: different combined features, feature-coding algorithms, and different data modality-based approaches. For each comparison, the parameter setting was provided with the reported results on the two public datasets. The confusion matrix analysis was also conducted for the proposed approach.

All the experiments were performed on a computer with an 11th Gen Intel(R) Core(TM) i7-1165G7 @ 2.80GHz CPU and Windows 11 Professional edition operating system using Matlab 2018b software.

### 4.2. Parameter Selection

Following the parameter selection scheme of FDDL and LCKSVD, we evaluated all parameters by using the five-fold cross-validation. There were four parameters in the proposed approach that needed to be adjusted, namely, the size of codebook M, parameter K for the K-means clustering algorithm, regularization parameter c for linear SVM, and the segmentation of subregions for SPM. A codebook with 1024 bases was pre-trained for the two datasets and three-level 4 × 4, 2 × 2, and 1 × 1 subregions were used for SPM. Therefore, the dimensions of the final feature vectors were 21,504 according to Equation (7). According to [35,36], two trade-off parameters λ_1_ and λ_2_ of FDDL and four parameters (dictionary size, sparsity, and two trade-off parameters α and β) of LCKSVD were set. The parameter selections of the benchmark approaches are summarized in Table 1.

### 4.3. Experimental Results and Comparative Analysis on Weizmann Dataset

#### 4.3.1. Comparison of Different Feature Combinations

We evaluated the proposed feature extraction strategy with several existing feature combinations, which contained MHI+BoW [7], MEI+PHOG [41], MHI+PHOG [41], MEI+R [41], and MHI+R [41]. An SVM classifier with a linear kernel function was employed for the aforementioned feature combinations, except for MHI+BoW [7], which used a KNN classifier. The results of different combined features on the Weizmann dataset are shown in Table 2.

The proposed feature combinations (i.e., with HPD) obtained better recognition results, and the accuracy was substantially higher than the other methods. These results demonstrate that the combination of energy image species and HPD is an effective strategy, as it combines the global features and local features to better describe human behavior for recognition.

#### 4.3.2. Comparison of Feature-Coding Algorithms

The ALLC algorithm was evaluated by comparing it against two other state-of-the-art feature-coding algorithms: Fisher discrimination dictionary learning (FDDL) [42] algorithm and label consistent K-SVD (LCKSVD) [43] algorithm. To get reliable results under different features, we need to indicate that if the subregions segmentation parameter for SPM is set to be 0, i.e., l=0, means that the original energy image species would not be segmented, and the MEI+HPD and MHI+HPD features will be reduced to MEI and MHI features, respectively. Therefore, we compared the ALLC algorithm with the aforementioned two feature-coding methods under the same features. The testing results of the feature-coding algorithm comparison on the Weizmann dataset are shown in Table 3.

Obviously, the performance of the ALLC algorithms was comparable to the other two feature-coding algorithms: FDDL and LCKSVD. For example, considering the MEI feature, the accuracy of the ALLC algorithm was 95.06%, while the accuracy of FDDL, LCKSVD1, and LCKSVD2 were 96.3%, 92.6%, and 95.07%, respectively. There were similar results for the energy image species MHI. These results prove that the ALLC feature-coding method can achieve a comparable result while being more computationally efficient.

#### 4.3.3. Comparison with Other Behavior Recognition Approaches

The evaluation results of the proposed approach with other existing approaches, including 3D-SIFT [10], HOGS [11], and HOG+CNN [24] are summarized in Table 4.

Referring to Table 4, we can see that the accuracy of MHI+HPD+ALLC was 98.77%, which is a little lower than HOGS [11] and HOG+CNN [24] with an accuracy of 99.65% and 99.4%, respectively. However, the proposed approach of MEI+HPD+ALLC achieved the highest accuracy of 100%. Thus, the proposed approach is comparable with the existing state-of-the-art approaches, especially with small-scale datasets, such as the Weizmann dataset. Here, an exciting result is that the proposed approach reached a comparable accuracy to the HOG+CNN approach [24]. This indicates that the proposed approach can offer comparable results to CNN-based approaches in targeted behavior recognition scenarios. Literature [24] also indicated that the training/testing ratio gives scope for a significant role in achieving greater accuracy; it reported that a 70:30 (70: training, 30: testing) ratio is considered optimal, however, with 80:20 and 50:50, the results tend to reduce. Therefore, CNN-based approaches are sensitive to the training/testing ratio. In comparison, our approach does not need to consider the training/testing ratio more.

### 4.4. Experimental Results and Comparative Analysis on DHA Dataset

#### 4.4.1. Comparison of Different Feature Combinations

The proposed feature extraction strategy was compared with different combined features, which contain HOGS [11], depth multi-perspective projections and PHOG features (DMPP+PHOG) [13], depth-limited RGB multi-perspective projection and PHOG features (DLRMPP+PHOG) [13], fusion of the RGB and depth features of DMPP and DLRMPP (DMPP+DLRMPP+PHOG) [13], GIST feature combined with space–time interest points from depth videos (GIST+DSTIPs) [21], and human pose representation model and temporal modeling representation (HPM+TM) [22]. The comparison results of different feature combinations on the DHA dataset are shown in Table 5.

From Table 5, one can see that, for RGB data modality, the HOGS [11] feature has achieved the highest recognition rate 99.39%. The proposed approach with 3 different energy image species (AMEI+HPD, EMEI+HPD, and MEnI+HPD) achieves a comparable recognition rate between 95% and 97%. The results further prove that the proposed strategy of combing energy image species and HPD can represent the human behavior well for recognition.

#### 4.4.2. Comparison of Feature-Coding Algorithms

The ALLC algorithm was evaluated and compared with four other existing feature-coding algorithms: SRC, CSR, FDDL, and LCKSVD. We also need to indicate that the subregions segmentation parameter for SPM was also set to be 0, i.e., l=0, and the three different combined features (AMEI+HPD, EMHI+HPD, and MEnI+PHD) will reduce to the original energy image species (AMEI, EMHI, and MEnI). The results of the feature-coding algorithm comparison on the DHA dataset are detailed in Table 6.

Taking MEnI+HPD features, the proposed approach can achieve an improvement of 1% to 4% compared with most of the benchmark methods and also achieves a comparable result with the HPM+TM approach.

#### 4.4.3. Comparison of Different Multi-Modality Fusion Methods

RGB is an essential channel of RGB-D data, which includes rich information features, e.g., color, shape, and texture. While depth images could provide information about the distance from the surface of the scene object of the viewpoint. Aiming to get higher accuracy and robustness in human behavior recognition, many researchers focus on depth-modality data-based approaches and multi-modality data-based approaches. Even the proposed approach mainly targets the RGB data, and was evaluated against some competitive single modality (RGB or depth)-based approaches and multimodality-based approaches. The testing results of different modality data-based approaches on the DHA dataset are summarized in Table 7.

The proposed approach achieved better performance compared to the depth modality data-based approach, with about 2–7% improvement in recognition accuracy. Compared with RGB modality data-based approaches, it was better than HPM+TM [22] and DLRMPP+PHOG [13], but had a little lower accuracy than HOGS [11]. In comparison with the multi-modality fusion approaches, DMPP+DLRMPP+PHOG [13] and HPM+TM+ CSR [18] obtained marginally higher accuracy of 98.2% and 98.6%, respectively, while MMDJM_GIST_DSTIP [21] and HPM+TM+SRC [22] achieved a slightly lower recognition accuracy than the proposed approaches.

It is worth mentioning that in Table 3 and Table 5, Table 6 and Table 7 the results consist of three parts. For FDDL [42] and LC-KSVD [43], we implemented the publicly available code provided by the authors on the datasets. For HOGS [11], GIST+DSTIPs [20], and HPM+TM [21], the results are cited directly from their original work. The rest are the results of the proposed methods.

#### 4.4.4. Confusion Matrix Analysis

**Figure 7:**
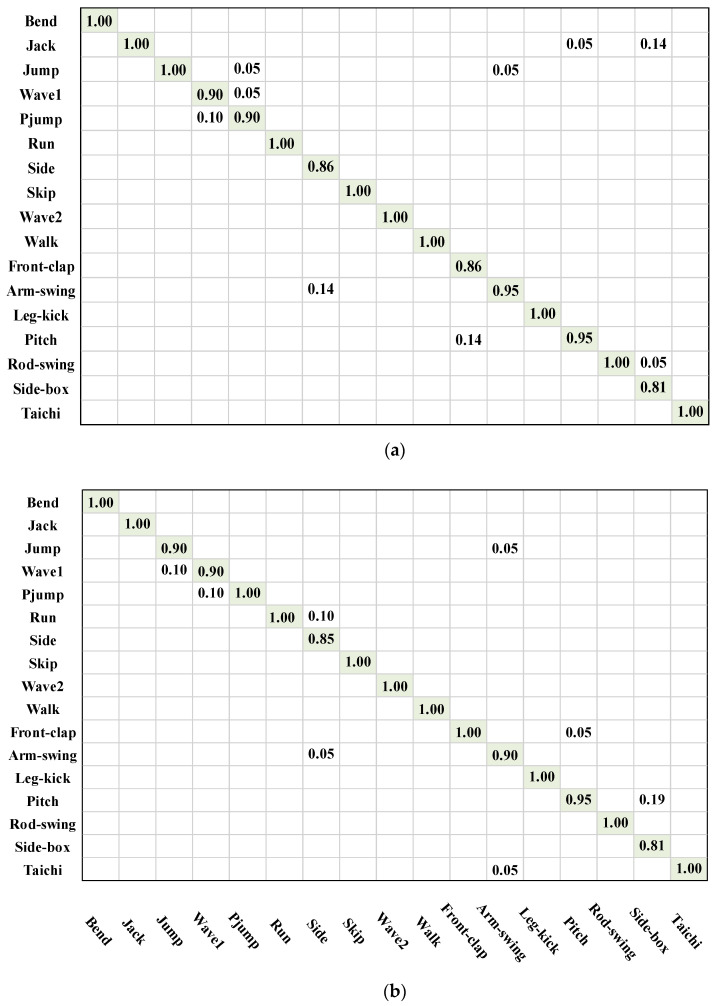
The confusion matrices for different energy image species descriptions. (**a**) Confusion matrix of DHA dataset based on AMEI and (**b**) confusion matrix of DHA dataset based on EMEI.

To make further analyses of the recognition performance, a correlation analysis was carried out by using the confusion matrix. In this section, the confusion matrices of two energy image species (AMEI and EMEI) are presented in Figure 7a,b, respectively. According to the confusion matrices and analysis results, the following conclusions can be drawn:

(1) The lowest correct recognition rate was 81% for both AMEI and EMEI on the DHA dataset; 10 and 11 out of 17 types of behaviors achieved 100% accuracy in recognition, respectively.
(2) Through analysing the confusion matrix, we can observe that certain behaviors were similar and may have caused confusion with each other; for example, wave1 and pjump; skip and jump; walk, skip and run; wave2 and leg-kick; pjump and jump; arm-swing and tai chi; side-box, jack, and pitch. Especially for side-box behavior, owing to the different motion ranges, angles, and boxing directions of the different performers, the accuracy was only 81%.
(3) For behaviors with high similarity and involving position change, such as run, pjump, front-clap, side, the recognition results were worse than the other behaviors. One possible reason is that those behaviors all contain leg and arm movements, however, their motion directions and positions may vary between image frames. Although HPD was constructed based on different energy image species for obtaining detailed motion features, they could not describe the depth information well. Therefore, it was difficult to identify these types of behaviors correctly.

## 5. Discussion

From Section 4.3 and Section 4.4, the experimental results prove that the proposed energy image species combined with the HPD feature extraction approach can better describe human behavior information than classical methods, because it describe the local and global features together. Meanwhile, the ALLC algorithm is a fast coding method, superior than the multi-modality algorithms which are computationally more expensive. One possible reason is that it has an analytical solution, thus it is more computationally efficient than some competitive feature-coding algorithms and multi-modality fusion approaches. Meanwhile, through sharing local bases, the ALLC algorithm could obtain the correlations between descriptors with similarity and make sure that patches with higher similarity have similar codes, which is very beneficial for feature recognition.

The research conducted in this work benefits other researchers that require automatic and robust extraction of self-learning features for human behavior recognition from video sequences in different ambient intelligence applications. Thus, it leads to us assume that this field may also quickly and effectively achieve good results in the case of insufficient data. However, there are still certain behaviors that usually contain depth information with a high degree of similarity, and the HPD could not describe the depth of information well.

## 6. Conclusions

Overall, many studies have been done on dictionary-learning-based approaches to human behavior recognition, and the present work adds other unique architectures involving energy images, the hierarchical patches descriptor (HPD), and the approximate locality-constrained linear coding (ALLC) algorithm. Experimental results and comparative analyses using the Weizmann and DHA datasets were demonstrated to be superior to some state-of-the-art approaches. In future work, to improve the robustness, we will consider constructing a human behavior model by fusing the RGB and depth information. In addition, in the case of large-scale data, deep-learning-based approaches need to be considered, such as multi-modality-based improved CNN and RNN.

## Figures and Tables

**Table 1:** Parameters selection of different approaches.

Approach	Ours				FDDL		LCKSVD			
Parameters	*M*	*K*	*c*	*L*	*λ*_1_	*λ*_2_	Dictionary Size	Sparsity	*α*	*β*
MEI	1024	5	7	2	0.05	0.5	60	8	0.05	0.001
MHI	1024	3	13	2	0.05	0.5	60	8	0.01	0.001
MEnI	1024	3	10	2	0.05	0.5	150	10	0.01	0.001
AMEI	1024	3	13	2	0.005	0.05	-	-	-	-
EMEI	1024	3	13	2	0.005	0.05	-	-	-	-

**Table 2:** Testing results of different combined feature comparisons on the Weizmann dataset.

Features	Accuracy Rate (%)
MHI+BoW [7]	90
MEI+PHOG [41]	82.7
MHI+PHOG [41]	92.6
MEI+R [41]	86.4
MHI+R [41]	81.5
Our MEI+HPD	100
Our MHI+HPD	98.77

**Table 3:** Testing results of feature-coding algorithm comparison on the Weizmann dataset.

Features	Feature-Coding Algorithms	Accuracy Rate (%)
MEI	LCKSVD1	92.6
MEI	LCKSVD2	95.07
MHI	LCKSVD1	93.83
MHI	LCKSVD2	96.3
MEI	FDDL	96.3
MHI	FDDL	95.06
MEI	Our ALLC	95.06
MHI	Our ALLC	93.83

**Table 4:** Testing results of some competitive approaches on the Weizmann dataset.

Features	Classifiers	Accuracy Rate (%)
3D-SIFT [10]	KNN	97.84
HOGS [11]	KNN	99.65
HOG+CNN [24]	SVM	99.4
Our MEI+HPD+ALLC	SVM	100
Our MHI+HPD+ALLC	SVM	98.77

**Table 5:** Testing results of different feature combination comparisons on the DHA dataset.

Features	Accuracy Rate (%)
HOGS [11]	99.39
DMPP+PHOG [13]	95
DLRMPP+PHOG [13]	95.6
DMPP+DLRMPP+PHOG [13]	98.2
GIST+DSTIPs [21]	93
HPM+TM [22]	90.8
Our AMEI+HPD	95.52
Our EMEI+HPD	96.08
Our MEnI+HPD	97.61

**Table 6:** Testing results of feature coding algorithm comparison on the DHA dataset.

Features	Feature-Coding Algorithms	Accuracy Rate (%)
GIST+DSTIPs [17]	SRC	93
HPM+TM [18]	SRC	93
HPM+TM [18]	CSR	98.6
AMEI	FDDL	89.09
EMEI	FDDL	91.32
MEnI	LCKSVD1	92.88
MEnI	LCKSVD2	94.58
Our AMEI+HPD	Our ALLC	93.28
Our EMEI+HPD	Our ALLC	94.68
Our MEnI+HPD	Our ALLC	95.92

**Table 7:** Testing results of different modality data-based approaches on the DHA dataset.

Data Modality	Features	Accuracy Rate (%)
RGB	HOGS [11]	99.39
RGB	DLRMPP+PHOG [13]	95.6
RGB	HPM+TM [22]	91.9
RGB	Our AMEI+HPD	95.52
RGB	Our EMEI+HPD	96.08
RGB	Our MEnI+HPD	97.61
Depth	DMPP+PHOG [13]	95
Depth	GIST+DSTIPs [21]	94
Depth	HPM+TM [22]	90.8
RGB+Depth	DMPP+DLRMPP+PHOG [13]	98.2
RGB+Depth	MMDJM+GIST+DSTIP [21]	97
RGB+Depth	HPM+TM+CSR [22]	98.6
RGB+Depth	HPM+TM+SRC [22]	94.4

## Data Availability

Data will be made available on demand.
